# A Comparison of the Psycholinguistic Styles of Schizophrenia-Related Stigma and Depression-Related Stigma on Social Media: Content Analysis

**DOI:** 10.2196/16470

**Published:** 2020-04-21

**Authors:** Ang Li, Dongdong Jiao, Xiaoqian Liu, Tingshao Zhu

**Affiliations:** 1 Department of Psychology Beijing Forestry University Beijing China; 2 Institute of Psychology Chinese Academy of Sciences Beijing China; 3 National Computer System Engineering Research Institute of China Beijing China

**Keywords:** stigma, schizophrenia, depression, psycholinguistic analysis, social media

## Abstract

**Background:**

Stigma related to schizophrenia is considered to be the primary focus of antistigma campaigns. Accurate and efficient detection of stigma toward schizophrenia in mass media is essential for the development of targeted antistigma interventions at the population level.

**Objective:**

The purpose of this study was to examine the psycholinguistic characteristics of schizophrenia-related stigma on social media (ie, Sina Weibo, a Chinese microblogging website), and then to explore whether schizophrenia-related stigma can be distinguished from stigma toward other mental illnesses (ie, depression-related stigma) in terms of psycholinguistic style.

**Methods:**

A total of 19,224 schizophrenia- and 15,879 depression-related Weibo posts were collected and analyzed. First, a human-based content analysis was performed on collected posts to determine whether they reflected stigma or not. Second, by using Linguistic Inquiry and Word Count software (Simplified Chinese version), a number of psycholinguistic features were automatically extracted from each post. Third, based on selected key features, four groups of classification models were established for different purposes: (a) differentiating schizophrenia-related stigma from nonstigma, (b) differentiating a certain subcategory of schizophrenia-related stigma from other subcategories, (c) differentiating schizophrenia-related stigma from depression-related stigma, and (d) differentiating a certain subcategory of schizophrenia-related stigma from the corresponding subcategory of depression-related stigma.

**Results:**

In total, 26.22% of schizophrenia-related posts were labeled as stigmatizing posts. The proportion of posts indicating depression-related stigma was significantly lower than that indicating schizophrenia-related stigma (χ^2^_1_=2484.64, *P*<.001). The classification performance of the models in the four groups ranged from .71 to .92 (F measure).

**Conclusions:**

The findings of this study have implications for the detection and reduction of stigma toward schizophrenia on social media.

## Introduction

Stigma is a destructive phenomenon that undermines efforts to improve mental health and well-being in people with mental illness. A high perception of stigma is associated with reduced self-disclosure to psychotherapists and others, leading to delayed treatment. Schizophrenia is among the most stigmatized mental illnesses; it can be considered a paradigm for mental illness [1,2]. Therefore, it is essential to maintain schizophrenia-related stigma as the main focus of antistigma campaigns [3-5].

Enhancing public knowledge and beliefs about mental illnesses is foundational for stigma reduction. Because information distributed through mass media is an important contributor to the dissemination of materials that may increase mental illness stigma [6-11], mass media campaigns, especially social media campaigns, are effective in raising public awareness of mental health literacy. Social media enables users to bring personal experience into the public domain with the potential to influence public perceptions of mental illness [12]. It also allows users to create their own social networks, which can be leveraged to facilitate the acceptance of received knowledge and then accelerate changes in individual attitudes and behaviors [13-15]. However, such campaigns should be carefully planned and target knowledge deficits, suggesting a need for monitoring mental illness–related stigma on social media. To address this concern, a number of content analysis studies have been performed by human coders on social media to examine stigma related to different mental illnesses, including schizophrenia, depression, suicide, eating disorder, and obsessive-compulsive disorder [2,16-19]. However, the sheer volume of information available on social media makes it difficult for human coders to keep track of all information. For example, in China, Sina Weibo (a free social media site that is similar to Twitter) has over 500 million registered users and produces more than 100 million microblogs (Weibo posts) per day. Therefore, in order to develop more effective strategies to challenge stigma, there is a need for accurate and efficient detection of mental illness–related stigma on social media.

The way people use words provides insight into their psychological profiles [20]. By using computerized text analysis tools (eg, Linguistic Inquiry and Word Count [LIWC]), psycholinguistic analysis can be automatically performed on texts to characterize language use patterns in terms of psychologically meaningful categories, making it easier to keep track of social media data. Recent studies have concluded that psycholinguistic analysis methods can be used to discover characteristics of stigmatizing expressions in social media posts (eg, suicide- and depression-related stigma) [21,22]. However, to date, no research has investigated psycholinguistic characteristics of stigma related to schizophrenia. To our knowledge, there is no convincing evidence for the lack of psycholinguistic differences in the expression of stigma across different mental illnesses. Therefore, additional analysis is necessary to examine the ways in which stigma associated with schizophrenia is presented on social media.

To address this concern, this study aims to investigate psycholinguistic characteristics of schizophrenia-related stigma on social media (ie, Sina Weibo), and then attempts to explore whether schizophrenia-related stigma can be distinguished from stigma related to other mental illnesses (ie, depression-related stigma). Stigma associated with depression was selected for the comparison since it is considered to be one of the most stigmatized mental illnesses, particularly among social media users [2,21].

## Methods

### Overview

This protocol was reviewed and approved by the Institutional Review Board at the Institute of Psychology, Chinese Academy of Sciences. Participant consent was not obtained, as it is not required for analyzing publicly available data [17,18,21,22]. To protect the privacy of participants, personally identifiable information (including usernames, real names, and other personal information) was excluded from data analysis.

The research process included the following three steps: (a) data collection, (b) data preprocessing, and (c) data modeling.

### Data Collection

First, a participant pool was created for data collection. Similar to Twitter, Sina Weibo is a free social media site that enables users to communicate and interact with others by posting digital messages (Weibo posts). Although some users opt to privatize their accounts, the majority of Weibo posts are publicly available for viewing and downloading. According to a previous study [23], a total of 1,953,485 Sina Weibo users were identified as potential participants. However, to due restrictions on data access imposed by Sina Weibo, of these potential participants, Weibo posts from only 1.06 million users were available for download.

Second, data from these participants were downloaded to construct a database of Weibo posts. By using an application programming interface (API), on April 2012, Weibo posts were downloaded automatically from 1.06 million users since the beginning of their registration. After that, the database of Weibo posts was maintained and updated regularly, with the latest update occurring in June 2017.

Third, relevant Weibo posts were identified from the database. To identify posts that are highly relevant to the topics of schizophrenia and depression, all downloaded posts should be searched using schizophrenia- and depression-related keywords, respectively. In this study, in order to make sure that identified posts refer to schizophrenia or depression as mental illnesses, two sets of keywords were selected, including “depressive disorder” (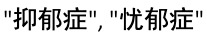) and “schizophrenia” (
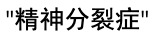
). Therefore, posts with such keywords were included for further analysis. A total of 15,879 depression-related posts were obtained from 10,130 distinct users (time period: September 21, 2009, to June 9, 2017); a total of 19,224 schizophrenia-related posts were obtained from 15,676 distinct users (time period: September 24, 2009 to June 11, 2017). More details about the gender and location of these users can be found in Table 1.

**Table 1:** Demographics of included users.

Characteristic		Users, depression-related posts (n=10,130), n (%)	Users, schizophrenia-related posts (n=15,676), n (%)
**Gender**			
	Male	3208 (31.67)	7152 (45.62)
	Female	6457 (63.74)	8425 (53.74)
	Not specified	465 (4.59)	99 (0.63)
**Location (provinces)**			
	Anhui	97 (0.96)	187 (1.19)
	Aomen	13 (0.13)	24 (0.15)
	Beijing	1138 (11.23)	2039 (13.01)
	Chongqing	143 (1.41)	205 (1.31)
	Fujian	283 (2.79)	458 (2.92)
	Gansu	27 (0.27)	66 (0.42)
	Guangdong	1732 (17.10)	2789 (17.79)
	Guangxi	134 (1.32)	201 (1.28)
	Guizhou	62 (0.61)	115 (0.73)
	Hainan	31 (0.31)	78 (0.50)
	Hebei	122 (1.20)	217 (1.38)
	Henan	208 (2.05)	285 (1.82)
	Heilongjiang	86 (0.85)	124 (0.79)
	Hong Kong	46 (0.45)	121 (0.77)
	Hubei	206 (2.03)	323 (2.06)
	Hunan	157 (1.55)	241 (1.54)
	Inner Mongolia	40 (0.39)	74 (0.47)
	Jilin	64 (0.63)	86 (0.55)
	Jiangsu	421 (4.16)	680 (4.34)
	Jiangxi	68 (0.67)	111 (0.71)
	Liaoning	185 (1.83)	273 (1.74)
	Ningxia	19 (0.19)	38 (0.24)
	Qinghai	6 (0.06)	8 (0.05)
	Shandong	752 (7.42)	1146 (7.31)
	Shanxi	63 (0.62)	122 (0.78)
	Shaanxi	134 (1.32)	235 (1.50)
	Shanghai	1073 (10.59)	1524 (9.72)
	Sichuan	271 (2.68)	489 (3.12)
	Taiwan	18 (0.18)	55 (0.35)
	Tianjin	125 (1.23)	180 (1.15)
	Tibet	11 (0.11)	16 (0.10)
	Xinjiang	49 (0.48)	61 (0.39)
	Yunnan	80 (0.79)	161 (1.03)
	Zhejiang	555 (5.48)	740 (4.72)
	International	634 (6.26)	1112 (7.09)
	Not specified	1077 (10.63)	1092 (6.97)

### Data Preprocessing

After data collection, data preprocessing was performed on raw data to prepare it for data modeling.

First, to obtain predicted class labels for data modeling, a content analysis was performed on collected Weibo posts to determine whether they reflected stigma or not. The coding framework was developed on the basis of expert consensus and available evidence. Specifically, a researcher reviewed relevant studies [17,24-27], and conducted an inductive analysis of all collected posts to construct an initial framework. After that, two human coders were recruited and received training on the content of the initial framework and gave suggestions for its amendment. Finally, the initial framework was amended accordingly, and the formal framework was established. As a result, the formal framework of schizophrenia-related stigma included 11 subcategories while the formal framework of depression-related stigma included 9 subcategories [21]. There was a high degree of overlap in stigma subcategories between the two frameworks. Specifically, the framework of schizophrenia-related stigma was quite similar to that of depression-related stigma, but two additional subcategories (ie, weird and stupid) were added to make a set of eleven. By using the formal frameworks, two independent human coders were instructed to analyze all Weibo posts with keywords (15,879 depression-related posts and 19,224 schizophrenia-related posts). Levels of consistency of coding were evaluated by computing Cohen kappa coefficients. If cases of inconsistency, input from a third researcher was used to resolve the issue. The coding results were considered as the ground truth for data modeling validation.

Second, to obtain predictors for data modeling, LIWC software (Simplified Chinese version) was used to automatically extract psycholinguistic features from each post. This is a reliable and valid text analysis tool for the automatic estimation of word frequency in multiple psychologically meaningful categories, including linguistic processes (eg, personal pronouns), psychological processes (eg, affective processes), personal concerns (eg, achievement), and spoken categories (eg, assent) [28]. For example, values of features indicating personal pronouns, affective processes, achievement, and assent refer to frequencies of words associated with personal pronouns (eg, them), affective processes (eg, abandon), achievement (eg, hero), and assent (eg, agree), respectively. To remove the effects of keywords on data modeling, keywords, including “depressive disorder” and “schizophrenia”, were deleted from each post before feature extraction. Therefore, a number of psycholinguistic features can be obtained for each post. Finally, standardized values of psycholinguistic features were computed as potential predictors.

### Data Modeling

In this study, the Waikato Environment for Knowledge Analysis software (Weka, version 3.8.1) was used to build four groups of classification models.

The first group of classification models was built to differentiate schizophrenia-related stigma from nonstigma. To solve the class imbalance problem, a certain number of posts were randomly selected from the majority class to obtain a well-balanced data set. After that, to improve the performance of data modeling, psycholinguistic features that were valid for differentiating between posts with and without stigma toward schizophrenia were selected as key features. Specifically, for each psycholinguistic feature, an independent sample *t* test was performed to compare values between two groups (schizophrenia-related stigma and nonstigma), and then the effect size value (Cohen *d*, which is one of the most common ways to measure effect size and can be used to indicate the standardized difference between two means) was calculated using the estimated *t* value. In this study, features that were statistically significant at .05 and had a Cohen *d* >0.20 or <–0.20 were considered as key features. Finally, by using four different algorithms (support vector machine [SVM]; naïve Bayes [NB]; multilayer perceptron neural network [MPNN]; logistic model trees [LMT]), four classification models (SVM, NB, MPNN, and LMT models) were established based on selected key features. Each model was tested by 10-fold cross-validation. Specifically, the data set was randomly divided into ten subgroups with the same sample size. Each subgroup was used to test the model that was built on the other nine subgroups. After 10 rounds of model training, the modeling results were integrated into a final model. The classification performance was evaluated by three indicators: precision (number of true positives / number of instances predicted to be positive), recall (number of true positives / number of positive instances), and F measure (a tradeoff between precision and recall).

The second group of classification models was built to differentiate a certain subcategory of schizophrenia-related stigma from other subcategories. In this study, two major subcategories of stigma related to schizophrenia (unpredictable and dangerous stigma) were examined (unpredictable stigma / other subcategories; dangerous stigma / other subcategories). The balanced classification data sets, key features, and classification models were obtained using the same method outlined above.

The third group of classification models was built to differentiate schizophrenia-related stigma from depression-related stigma. The balanced classification data set, key features, and classification models were obtained using the same method outlined in the section on the first group of classification models.

For the third group of classification models, the reason for good classification performance may be attributed to marked differences in amount and distribution of stigma subcategories between schizophrenia and depression rather than actual existence of differences in psycholinguistic style between schizophrenia- and depression-related stigma. To clarify this issue, the fourth group of classification models was built to differentiate a certain subcategory of schizophrenia-related stigma from the corresponding subcategory of depression-related stigma. To obtain enough data for further analysis in this study, two subcategories of stigma (unpredictable and glorified stigma) were examined (unpredictable stigma related to schizophrenia / unpredictable stigma related to depression; glorified stigma related to schizophrenia / glorified stigma related to depression). The balanced classification data sets, key features, and classification models were obtained using the same method outlined in the section on the first group of classification models.

## Results

### Coding

The coding results for stigma related to schizophrenia are shown in Table 2 (see Multimedia Appendix 1 for posts in Chinese). The Cohen kappa coefficients for schizophrenia-related stigma and its subcategories reached .77 and .78, respectively, reflecting a satisfying level of agreement [29]. Of all schizophrenia-related posts, 26.22% (5041/19,224) were labeled as stigmatizing posts. Of these posts, 41.14% (n=2074) and 26.86% (n=1354) reflected the view that “people with schizophrenia are unpredictable” (unpredictable stigma) and “people with schizophrenia are dangerous” (dangerous stigma), respectively.

**Table 2:** Coding framework for schizophrenia-related stigma.

Subcategory	Definition	Representative Weibo post (English translation)	Posts, n (%)
It is best to avoid people with schizophrenia so that you do not become vulnerable to schizophrenia	Beliefs that schizophrenia is an infectious disease	“It is said that schizophrenia is highly infectious...”	26 (0.52)
Schizophrenia is a sign of personal weakness	Beliefs that people with schizophrenia show a lack of strength and cannot sustain pressure	“...A mentally weak person is so vulnerable to schizophrenia...”	39 (0.77)
People with schizophrenia are dangerous	Beliefs that people with schizophrenia are likely to cause harm or injury	“When you need to talk to a person with schizophrenia, it is very important to pay attention to your safety!”	1354 (26.86)
People with schizophrenia are unpredictable	Beliefs that people with schizophrenia behave in a way that cannot be not easily predicted	“People with schizophrenia can suddenly turn crying into laughing (still with snots and tears of sadness)...”	2074 (41.14)
Schizophrenia is not a real medical illness	Beliefs that schizophrenia is a made up, rather than a medical disease	“Schizophrenia is really just an excuse to get a lesser sentence or to get out of prison”	21 (0.42)
People with schizophrenia could snap out of it if they wanted	Beliefs that people with schizophrenia can recover from their illness at will	“As you pray to the Buddha, your schizophrenia will be recovered soon...”	13 (0.26)
People would not tell anyone if they had schizophrenia	Beliefs that people should be ashamed of their own schizophrenia	“...Schizophrenia is God's punishment for family sins...”	115 (2.28)
People with schizophrenia are glorified	Beliefs that schizophrenia is a sign of noble souls or a quality of being graceful	“I think those with schizophrenia are charming...”	281 (5.57)
People with schizophrenia are self-centered	Beliefs that people with schizophrenia only think of their own advantage	“...Schizophrenia is the same as narcissism.”	48 (0.95)
People with schizophrenia are weird	Beliefs that people with schizophrenia behave in an unsettling way that is strikingly odd or unusual	“...Someone is throwing money like paper towels. Could this person have schizophrenia?”	929 (18.43)
People with schizophrenia are stupid	Beliefs that people with schizophrenia are silly or unwise	“The schizophrenia patients, the idiot people”	141 (2.80)

The coding results for stigma associated with depression showed that 6.09% (967/15,879) of depression-related posts were labeled as stigmatizing posts. Further details on coding results can be found in a previous study [21].

The proportion of posts indicating depression-related stigma (967/15,879) was significantly lower than that indicating schizophrenia-related stigma (5041/19,224) (*χ*^2^_1_=2484.64, *P*<.001).

### Differentiating Schizophrenia-Related Stigma From Nonstigma

A total of 13 key features were selected for data modeling (Multimedia Appendix 2). Within each predicted class (schizophrenia-related stigma and nonstigma), there existed 5041 posts. The LMT model had the best classification performance (precision=.89, recall=.89, F measure=.89) (Table 3).

**Table 3:** Performance of classification models.

Models		Stigma / nonstigma	Unpredictable / other subcategories	Dangerous / other subcategories	Depression / schizophrenia	Unpredictable (depression) / unpredictable (schizophrenia)	Glorified (depression) / glorified (schizophrenia)
**Support vector machine**							
	Precision	.70	.72	.76	.80	.93	.65
	Recall	.70	.72	.76	.79	.92	.65
	F measure	.70	.72	.76	.79	.92	.65
**Naïve Bayes**							
	Precision	.65	.67	.69	.67	.88	.67
	Recall	.60	.64	.66	.65	.88	.65
	F measure	.57	.62	.64	.64	.88	.64
**Multilayer perceptron neural network**							
	Precision	.67	.69	.71	.75	.88	.71
	Recall	.67	.69	.71	.75	.88	.71
	F measure	.67	.69	.71	.75	.88	.71
**Logistic model trees**							
	Precision	.89	.70	.75	.78	.91	.65
	Recall	.89	.70	.75	.77	.91	.65
	F measure	.89	.70	.75	.77	.91	.65

### Differentiating a Certain Subcategory of Schizophrenia-Related Stigma From Other Subcategories

A total of 28 and 27 key features were selected for building two subgroups of classification models respectively: one for differentiating between unpredictable stigma and other subcategories, and one for differentiating between dangerous stigma and other subcategories (Multimedia Appendix 2). For the first subgroup, within each predicted class (unpredictable stigma and other subcategories), there existed 2074 posts, while for the second subgroup, within each predicted class (dangerous stigma and other subcategories), there existed 1354 posts. For both of the two subgroups, the SVM model had the best classification performance (subgroup 1: precision=.72, recall=.72, F measure=.72; subgroup 2: precision=.76, recall=.76, F measure=.76) (Table 3).

### Differentiating Schizophrenia-Related Stigma From Depression-Related Stigma

A total of 30 key features were selected for data modeling (Multimedia Appendix 2). Within each predicted class (depression- and schizophrenia-related stigma), there existed 967 posts. The SVM model had the best classification performance (precision=.80, recall=.79, F measure=.79) (Table 3).

### Differentiating a Certain Subcategory of Schizophrenia-Related Stigma From the Corresponding Subcategory of Depression-Related Stigma

A total of 52 and 19 key features were selected for building two subgroups of classification models, respectively: one for differentiating the expression of unpredictable stigma between depression and schizophrenia, and one for differentiating the expression of glorified stigma between depression and schizophrenia (Multimedia Appendix 2). For the first subgroup, within each predicted class (unpredictable stigma related to depression and that related to schizophrenia), there existed 380 posts, while for the second subgroup, within each predicted class (glorified stigma related to depression and that related to schizophrenia), there existed 114 posts. Furthermore, for the first subgroup, the SVM model had the best classification performance (precision=.93, recall=.92, F measure=.92), while for the second subgroup, the MPNN model had the best classification performance (precision=.71, recall=.71, F measure=.71) (Table 3).

## Discussion

### Principal Findings

According to our knowledge, this is the first study to investigate the psycholinguistic characteristics of schizophrenia-related stigma on social media and characterize psycholinguistic differences in the expression of stigma between two different mental illnesses (depression and schizophrenia). The findings of this study have a number of implications for the detection and reduction of stigma associated with schizophrenia on social media.

First, it is necessary for campaigns to reduce stigma associated with schizophrenia on social media. Results showed that schizophrenia-related stigma was prevalent on Chinese social media. According to this study, 26.22% of relevant Weibo posts indicated stigmatizing attitudes toward schizophrenia, which is higher than that reported by previous studies on Twitter (5%-9.7%) [17,30]. Such inconsistency may be partly due to the role of anonymity in increasing the likelihood of users to express stigmatizing attitudes on Sina Weibo (an anonymous social media site) rather than on Twitter (an open public forum), and partly due to a low level of schizophrenia literacy among people in China [31-33]. In addition, stigmatization of schizophrenia was significantly higher than stigmatization of depression (*χ*^2^_1_=2484.64, *P*<.001), suggesting the essential role of schizophrenia-related stigma in antistigma campaigns on social media. Moreover, people with schizophrenia were more frequently perceived as unpredictable and dangerous (41.14% and 26.86%), which should be the targets of stigma reduction campaigns. It is worth noting that these results are largely consistent with relevant studies [2,24,26,34-38], supporting the use of social media to monitor mental illness–related stigma.

Second, the use of psycholinguistic analysis methods facilitates automatic detection of stigma toward schizophrenia on social media. Results showed that, by using psycholinguistic analysis methods, the best performance for detecting schizophrenia-related stigma and its subcategories ranged from .72 to .89 (F measure). Compared with the results of other studies (F measure=.66 to .86) [21,22], this performance is satisfactory. It is worth noting that, in this study, no single algorithm achieved the best performance in all six classification tasks (Table 3). Although it is still unclear why the performance of algorithms varied considerably across tasks, the results of this study imply that it is necessary to select appropriate algorithms for solving different tasks.

More importantly, the use of psycholinguistic analysis methods may provide insight into the ways in which schizophrenia-related stigma is presented on social media. Multimedia Appendix 2 showed that the expression of schizophrenia-related stigma was associated with increased use of words related to social processes (eg, mate), humans (eg, adult), death (eg, kill), and anger (eg, hate). Such language use patterns indicate a preference for more social comparisons and a higher level of negative emotion, which may fit into two elements of stigma processes, including cognitive separation (comparisons between people with and without a stigmatizing label) and emotional reactions (negative emotional reactions to people with a stigmatizing label) [39]. In addition, there existed psycholinguistic differences between subcategories of schizophrenia-related stigma as well. For example, compared with other subcategories, the expression of unpredictable stigma was associated with more frequent use of words related to cognitive processes (eg, ought) and personal pronouns (eg, them), which may be due to sustained confusion about patient behavior and the misbelief that people with schizophrenia have multiple personalities. Unlike unpredictable stigma, the expression of dangerous stigma was associated with an increased use in words related to death (eg, kill) and health (eg, clinic), which may be because of the expectation that patients with schizophrenia are likely to cause harm or injury.

Third, the development of an accurate tool for measuring stigma should be disease-specific. Results showed that stigma associated with schizophrenia can be distinguished from depression-related stigma in terms of psycholinguistic style. These significant differences existed not only at the level of general stigma (depression- or schizophrenia-related stigma as a whole), but also at the level of stigma subcategories (eg, unpredictable and glorified stigma) (Multimedia Appendix 2). The classification performance of the corresponding models ranged from .71 to .92 (F measure) (Table 3). These results can be explained by the fact that the reason for good classification performance may not be solely attributed to differences in amount and distribution of stigma subcategories between schizophrenia and depression, but also to the actual existence of differences in psycholinguistic style between stigma related to schizophrenia and depression. Therefore, in order to improve stigma detection performance, it is necessary to develop disease-specific measurement tools.

### Limitations

There are a number of limitations. First, it is unknown whether all relevant posts can be searched by keywords that were used in this study. Therefore, it is unclear whether any additional language use patterns are associated with stigmatizing expressions. In addition, it is uncertain whether the current keywords selection may bias estimations of the number of stigmatizing posts. Second, social media users are not representative of the general population. For example, in China, Sina Weibo users are more likely to be aged between 20 to 29 years old, well educated, and located in urban areas [40]. Therefore, the findings of this study might have limited generalizability. Third, in this study, all analyzed social media posts were written in Chinese. Therefore, it is uncertain whether the findings of this study will be applicable to other languages. Fourth, although all established models were validated using the method of cross-validation, they should be further tested on other data sets in the future.

### Conclusions

In this study, a nonintrusive method was used to collect and analyze data under nonexperimental conditions. As a result, the current research should have high ecological validity and could investigate actual attitudes toward people with schizophrenia. The results of this study may facilitate the automatic detection of stigma on social media and improve social media campaigns related to stigma reduction.

## Appendix

Multimedia Appendix 1 Coding framework for schizophrenia-related stigma.

Multimedia Appendix 2 Key features selected for data modeling.

## Abbreviations

API: application programming interface
LIWC: Linguistic Inquiry and Word Count
SVM: support vector machine
NB: naïve Bayes
MPNN: multilayer perceptron neural network
LMT: logistic model trees
