# Supplementary material for: A Comparison of the Psycholinguistic Styles of Schizophrenia-Related Stigma and Depression-Related Stigma on Social Media: Content Analysis
Source: J Med Internet Res. 2020 Apr 21;22(4):e16470. doi: 10.2196/16470 (PMC7201321; doi:10.2196/16470)
Supplement: Multimedia Appendix 1 [file jmir_v22i4e16470_app1.pdf]

Multimedia Appendix 1. Coding framework for schizophrenia-related stigma.

Subcategory	Definition	Representative Weibo post	Posts, n (%)
It is best to avoid people with schizophrenia so that you do not become vulnerable to schizophrenia	Beliefs that schizophrenia is an infectious disease	"It is said that schizophrenia is highly infectious..." ["传说精神分裂症的传染力很强..."]	26 (0.52)
Schizophrenia is a sign of personal weakness	Beliefs that people with schizophrenia show a lack of strength and cannot sustain pressure	"...A mentally weak person is so vulnerable to schizophrenia..." ["...很容易得精神分裂症的，特别是玻璃心的孩纸们..."]	39 (0.77)
People with schizophrenia are dangerous	Beliefs that people with schizophrenia are likely to cause harm or injury	"When you need to talk to a person with schizophrenia, it is very important to pay attention to your safety!" ["采访严重精神分裂症患者要注意自身安全！"]	1354 (26.86)
People with schizophrenia are unpredictable	Beliefs that people with schizophrenia behave in a way that cannot be not easily predicted	"People with schizophrenia can suddenly turn crying into laughing (still with snots and tears of sadness) ..." ["精神分裂症病人，这一秒哭个不停，下一秒又傻笑出声（鼻涕眼泪还挂着）..."]	2074 (41.14)
Schizophrenia is not a real medical illness	Beliefs that schizophrenia is a	"Schizophrenia is really just an excuse to	21 (0.42)

	made up, rather than a medical disease	get a lesser sentence or to get out of prison" ["精神分裂症真是万能的脱罪藉口"]	
People with schizophrenia could snap out of it if they wanted	Beliefs that people with schizophrenia can recover from their illness at will	"As you pray to the Buddha, your schizophrenia will be recovered soon ..." ["唱念观世音菩萨圣号治愈严重精神分裂症..."]	13 (0.26)
People would not tell anyone if they had schizophrenia	Beliefs that people should be ashamed of their own schizophrenia	"...Schizophrenia is God's punishment for family sins..." ["...患精神分裂症，原来是祖上有杀业灵性所致..."]	115 (2.28)
People with schizophrenia are glorified	Beliefs that schizophrenia is a sign of noble souls or a quality of being graceful	"I think those with schizophrenia are charming..." ["从来没觉得精神分裂症有这么迷人过..."]	281 (5.57)
People with schizophrenia are self-centered	Beliefs that people with schizophrenia only think of their own advantage	"...Schizophrenia is the same as narcissism." ["...自恋狂、精神分裂症...是同一类人。"]	48 (0.95)
People with schizophrenia are weird	Beliefs that people with schizophrenia behave in an unsettling way that is strikingly odd or unusual	"...Someone is throwing money like paper towels. Could this person have schizophrenia?" ["...把钱当手纸丢了，这人不会得精神分裂症吧？"]	929 (18.43)

People with schizophrenia are stupid	Beliefs that people with schizophrenia are silly or unwise	"The schizophrenia patients, the idiot people" ["傻逼精神分裂症患者"]	141 (2.80)
