# Supplementary material for: A Comparison of the Psycholinguistic Styles of Schizophrenia-Related Stigma and Depression-Related Stigma on Social Media: Content Analysis
Source: J Med Internet Res. 2020 Apr 21;22(4):e16470. doi: 10.2196/16470 (PMC7201321; doi:10.2196/16470)
Supplement: Multimedia Appendix 2 [file jmir_v22i4e16470_app2.pdf]

Multimedia Appendix 2. Key features selected for data modeling.

Comparisons	Features	Example words	*t*	*df*	*P* value	Cohen *d*
Schizophrenia-related stigma vs Nonstigma	Social processes	mate	18.43	10080	<.001	0.37
	Time	until	17.85	10080	<.001	0.36
	Adverbs	very	17.50	10080	<.001	0.35
	Humans	adult	16.12	10080	<.001	0.32
	Total function words	/	15.89	10080	<.001	0.32
	Signal words for present tense	often	14.44	10080	<.001	0.29
	Death	kill	13.79	10080	<.001	0.28
	First pers plural	we	13.38	10080	<.001	0.27
	Conjunctions	but	13.38	10080	<.001	0.27
	Relativity	stop	12.81	10080	<.001	0.26
	Word count	/	12.56	10080	<.001	0.25
	Signal words for tenses	already	11.81	10080	<.001	0.24
	Anger	hate	11.22	10080	<.001	0.22
Unpredictable stigma (schizophrenia) vs Other subcategories (schizophrenia)	Personal pronouns	them	21.23	4146	<.001	0.66
	First pers singular	me	19.51	4146	<.001	0.61
	Total function words	/	17.83	4146	<.001	0.55
	Signal words for tenses	already	17.36	4146	<.001	0.54
	Signal words for continuous tense	yet	15.35	4146	<.001	0.48
	First pers plural	we	14.68	4146	<.001	0.46
	Adverbs	very	14.46	4146	<.001	0.45
	Signal words for present tense	often	13.93	4146	<.001	0.43
	Signal words for past tense	ago	13.01	4146	<.001	0.40
	Religion	altar	11.08	4146	<.001	0.34
	Assent	agree	11.00	4146	<.001	0.34
	Time	until	10.80	4146	<.001	0.34
	Conjunctions	but	10.56	4146	<.001	0.33
	Total pronouns	itself	10.24	4146	<.001	0.32
	Positive emotion	love	10.05	4146	<.001	0.31

	Cognitive processes	ought	9.89	4146	<.001	0.31
	Inclusive	with	9.28	4146	<.001	0.29
	Auxiliary verbs	will	7.33	4146	<.001	0.23
	Third pers singular	she	-7.09	4146	<.001	-0.22
	Impersonal pronouns	it	-7.33	4146	<.001	-0.23
	Space	down	-7.74	4146	<.001	-0.24
	Biological processes	pain	-8.38	4146	<.001	-0.26
	Body	hand	-8.77	4146	<.001	-0.27
	Family	daughter	-8.82	4146	<.001	-0.27
	Work	job	-8.84	4146	<.001	-0.27
	Word count	/	-8.92	4146	<.001	-0.28
	Motion	arrive	-9.12	4146	<.001	-0.28
	Death	kill	-14.07	4146	<.001	-0.44
Dangerous stigma (schizophrenia) vs Other subcategories (schizophrenia)	Death	kill	10.71	2706	<.001	0.41
	Family	daughter	10.04	2706	<.001	0.39
	Work	job	8.12	2706	<.001	0.31
	Third pers singular	she	6.49	2706	<.001	0.25
	Biological processes	pain	6.31	2706	<.001	0.24
	Health	clinic	6.14	2706	<.001	0.24
	Humans	adult	5.57	2706	<.001	0.21
	Fillers	I mean	-5.82	2706	<.001	-0.22
	Second pers singular	you	-6.00	2706	<.001	-0.23
	Auxiliary verbs	will	-6.19	2706	<.001	-0.24
	Common verbs	walk	-7.57	2706	<.001	-0.29
	Religion	altar	-7.67	2706	<.001	-0.29
	Signal words for past tense	ago	-8.33	2706	<.001	-0.32
	Conjunctions	but	-8.39	2706	<.001	-0.32
	Inclusive	with	-8.43	2706	<.001	-0.32
	Signal words for present tense	often	-8.53	2706	<.001	-0.33
	Positive emotion	love	-8.78	2706	<.001	-0.34
	First pers plural	we	-9.36	2706	<.001	-0.36
	Cognitive processes	ought	-10.94	2706	<.001	-0.42
	Assent	agree	-11.08	2706	<.001	-0.43
	Signal words for tenses	already	-11.29	2706	<.001	-0.43

	Signal words for continuous tense	yet	-11.36	2706	<.001	-0.44
	Total pronouns	itself	-11.82	2706	<.001	-0.45
	Adverbs	very	-12.13	2706	<.001	-0.47
	First pers singular	me	-14.33	2706	<.001	-0.55
	Personal pronouns	them	-14.57	2706	<.001	-0.56
	Total function words	/	-18.51	2706	<.001	-0.71
Depression-related stigma vs Schizophrenia-related stigma	Auxiliary verbs	will	15.59	1932	<.001	0.71
	Discrepancy	should	14.14	1932	<.001	0.64
	Cognitive processes	ought	13.54	1932	<.001	0.62
	Quantifiers	few	13.41	1932	<.001	0.61
	Tentative	maybe	12.76	1932	<.001	0.58
	Negative emotion	hurt	11.12	1932	<.001	0.51
	Affective processes	abandon	10.91	1932	<.001	0.50
	Conjunctions	but	10.36	1932	<.001	0.47
	Time	until	8.59	1932	<.001	0.39
	Inclusive	with	7.75	1932	<.001	0.35
	Second pers singular	you	7.18	1932	<.001	0.33
	Sad	crying	6.30	1932	<.001	0.29
	Prepositions	to	5.79	1932	<.001	0.26
	Adverbs	very	5.48	1932	<.001	0.25
	Total pronouns	itself	-4.69	1932	<.001	-0.21
	Words per sentence	/	-4.53	1932	<.001	-0.21
	Family	daughter	-4.77	1932	<.001	-0.22
	Signal words for present tense	often	-5.08	1932	<.001	-0.23
	Humans	adult	-5.08	1932	<.001	-0.23
	Positive emotion	love	-5.07	1932	<.001	-0.23
	Sexual	horny	-5.00	1932	<.001	-0.23
	Motion	arrive	-5.18	1932	<.001	-0.24
	Insight	think	-5.48	1932	<.001	-0.25
	Work	job	-6.08	1932	<.001	-0.28
	Religion	altar	-6.50	1932	<.001	-0.30
	First pers plural	we	-6.83	1932	<.001	-0.31
	Signal words for past tense	ago	-6.75	1932	<.001	-0.31
	Numbers	second	-7.09	1932	<.001	-0.32

	First pers singular	me	-7.80	1932	<.001	-0.35
	Space	down	-9.82	1932	<.001	-0.45
Unpredictable stigma (depression) vs Unpredictable stigma (schizophrenia)	Discrepancy	should	23.02	758	<.001	1.67
	Quantifiers	few	21.64	758	<.001	1.57
	Cognitive processes	ought	19.57	758	<.001	1.42
	Tentative	maybe	18.45	758	<.001	1.34
	Auxiliary verbs	will	15.68	758	<.001	1.14
	Negative emotion	hurt	15.40	758	<.001	1.12
	Affective processes	abandon	14.79	758	<.001	1.07
	Time	until	14.61	758	<.001	1.06
	Conjunctions	but	12.54	758	<.001	0.91
	Prepositions	to	11.73	758	<.001	0.85
	Inclusive	with	9.70	758	<.001	0.70
	Signal words for continuous tense	yet	7.91	758	<.001	0.57
	Relativity	stop	3.87	758	<.001	0.28
	Negations	never	-3.37	758	.001	-0.24
	Sexual	horny	-3.28	758	.001	-0.24
	Home	apartment	-3.34	758	.001	-0.24
	Swear	damn	-3.61	758	<.001	-0.26
	Ingestion	eat	-3.57	758	<.001	-0.26
	Common verbs	walk	-3.68	758	<.001	-0.27
	Family	daughter	-3.76	758	<.001	-0.27
	See	view	-3.94	758	<.001	-0.29
	Total function words	/	-4.26	758	<.001	-0.31
	Feel	touch	-4.72	758	<.001	-0.34
	Body	hand	-4.70	758	<.001	-0.34
	Second pers singular	you	-4.87	758	<.001	-0.35
	Signal words for future tense	tomorrow	-5.42	758	<.001	-0.39
	Fillers	I mean	-5.65	758	<.001	-0.41
	Inhibition	block	-5.74	758	<.001	-0.42
	Perceptual processes	observing	-5.83	758	<.001	-0.42
	Causation	because	-5.97	758	<.001	-0.43
	Health	clinic	-6.29	758	<.001	-0.46
	Impersonal pronouns	it	-6.81	758	<.001	-0.49
	Humans	adult	-6.78	758	<.001	-0.49

	Signal words for past tense	ago	-7.50	758	<.001	-0.54
	Motion	arrive	-7.56	758	<.001	-0.55
	Work	job	-7.53	758	<.001	-0.55
	Signal words for present tense	often	-7.75	758	<.001	-0.56
	First pers plural	we	-7.78	758	<.001	-0.57
	Insight	think	-8.05	758	<.001	-0.58
	Religion	altar	-7.99	758	<.001	-0.58
	Words per sentence	/	-8.11	758	<.001	-0.59
	Numbers	second	-8.38	758	<.001	-0.61
	Certainty	always	-8.42	758	<.001	-0.61
	Achievement	hero	-8.71	758	<.001	-0.63
	Positive emotion	love	-8.99	758	<.001	-0.65
	Biological processes	pain	-9.16	758	<.001	-0.67
	Word count	/	-10.46	758	<.001	-0.76
	Space	down	-11.82	758	<.001	-0.86
	Social processes	mate	-13.84	758	<.001	-1.01
	First pers singular	me	-14.19	758	<.001	-1.03
	Personal pronouns	them	-17.03	758	<.001	-1.24
	Total pronouns	itself	-19.43	758	<.001	-1.41
Glorified stigma (depression) vs Glorified stigma (schizophrenia)	Friends	buddy	4.35	226	<.001	0.58
	Second pers singular	you	4.20	226	<.001	0.56
	Discrepancy	should	3.81	226	<.001	0.51
	Certainty	always	2.68	226	.008	0.36
	Auxiliary verbs	will	2.59	226	.01	0.34
	Death	kill	2.59	226	.01	0.34
	Fillers	I mean	2.52	226	.01	0.34
	Time	until	2.19	226	.03	0.29
	Achievement	hero	-2.05	226	.04	-0.27
	Motion	arrive	-2.25	226	.03	-0.30
	Prepositions	to	-2.30	226	.02	-0.31
	Causation	because	-2.39	226	.02	-0.32
	Affective processes	abandon	-2.58	226	.01	-0.34
	Assent	agree	-2.60	226	.01	-0.35
	Signal words for tenses	already	-3.20	226	.002	-0.43

	Positive emotion	love	-3.26	226	.001	-0.43
	Signal words for past tense	ago	-4.35	226	<.001	-0.58
	Humans	adult	-5.49	226	<.001	-0.73
	Word count	/	-5.75	226	<.001	-0.76
